# RCA-Based Biosensor for Electrical and Colorimetric Detection of Pathogen DNA

**DOI:** 10.1186/s11671-016-1440-7

**Published:** 2016-05-04

**Authors:** Jaepil Jeong, Hyejin Kim, Dong Jun Lee, Byung Jun Jung, Jong Bum Lee

**Affiliations:** Department of Chemical Engineering, University of Seoul, Seoul, 130-743 South Korea; Department of Materials Science and Engineering, University of Seoul, Seoul, 130-743 South Korea

**Keywords:** Electrical detection, Rolling circle amplification, DNA detection, Biosensor, Multi-primer

## Abstract

For the diagnosis and prevention of diseases, a range of strategies for the detection of pathogens have been developed. In this study, we synthesized the rolling circle amplification (RCA)-based biosensor that enables detection of pathogen DNA in two analytical modes. Only in the presence of the target DNA, the template DNA can be continuously polymerized by simply carrying out RCA, which gives rise to a change of surface structure of Au electrodes and the gap between the electrodes. Electrical signal was generated after introducing hydrogen tetrachloroaurate (HAuCl_4_) to the DNA-coated biosensor for the improvement of the conductivity of DNA, which indicates that the presence of the pathogen DNA can be detected in an electrical approach. Furthermore, the existence of the target DNA was readily detected by the naked eyes through change in colors of the electrodes from bright yellow to orange-red after RCA reaction. The RCA-based biosensor offers a new platform for monitoring of pathogenic DNA with two different detection modes in one system.

## Background

The development of techniques for pathogen detection is crucial for the diagnosis and prevention of diseases from spreading. Indeed, a wide range of approaches were recently proposed for the rapid and sensitive detection of clinical pathogen deoxyribonucleic acid (DNA) from a classic polymerase chain reaction (PCR)-based system [1–3] to a surface-enhanced Raman scattering (SERS)-based technique [4–6]. For instance, a range of techniques have been reported for the rapid and quantitative analysis of avian influenza A virus which can cause a life-threatening respiratory illness [7, 8].

One of the approaches for detecting pathogenic DNAs is to exploit synthetic DNA structures, benefitting from high programmability of DNA. These DNA structures have been widely used as multifunctional building blocks. By taking advantage of programmable self-assembly, various shapes of DNA structures have been introduced for the pathogen detection [9–11].

In addition, technological advances in other fields have also accelerated the development of synthetic DNA structures [12–16]. Among a range of techniques, an enzymatic approach has benefitted a massive replication of DNA and efficient synthesis of DNA constructs [17–22]. In particular, rolling circle amplification (RCA) has been widely exploited because of its simple process and high efficiency in DNA polymerization [23]. RCA is an isothermal enzymatic technique which allows a constant generation of DNA complementary to circular template DNA. With a reliable reproducibility and target specificity, there has been enormous interest in using RCA for pathogenic diagnosis [24]. In many cases, however, RCA-based detection systems involve reporter molecules [25, 26] or gold nanoparticles [27, 28] for visualization of RCA products.

In this study, we demonstrated an RCA-based biosensor for pathogen DNA detection. RCA reaction was carried out after the synthesis of primer DNA-circular DNA (pri-cir DNA) hybridized complex on two gold electrodes with a gap. Only in the presence of the target DNA, the newly synthesized DNA strands bridged the gap between the electrodes. Importantly, multi-primer DNA was added at the beginning of RCA for an efficient DNA polymerization with highly branched DNA strands [29]. As a result, by applying voltages to the electrodes, generation of electrical signal was confirmed after simply incubating the substrate with hydrogen tetrachloroaurate (HAuCl_4_) without further reduction for metallization of DNA products. Discoloration of the electrodes was also observed in the presence of the target DNA without any reporter for recognition of the RCA products, which indicates that our biosensing system can be operated in both electrical and colorimetric manners.

## Methods

### Materials

All oligonucleotides (primer DNA: 5′-thiol-TTT TTT TTT TTT TTT TTT TTA CGA CGT GTG ACC ATG CA-3′; template DNA: 5′-ACT TGC GGC AAT ACA AGT CGT CTC GTC GCA CTC TTT TTG CAT GGT CAC ACG TCG TTC TAT TGT GCG ACG AGA CCG TTT CAA GAT CCC AAT GAT-3′; target DNA: 5′-TGT TAT TGC CGC AAG TAT CAT TGG GAT CTT GCA CTT-3′; multi-primer DNA: 5′-AT TGT GCG ACG AGA CCG T-3′) were purchased from Integrated DNA Technologies (USA). The target DNA was designed from influenza A virus sequence shown in GenBank database (http://www.ncbi.nlm.nih.gov/nuccore/401716582, accessed March 20, 2014). Thermally grown SiO_2_ on the Si wafer was purchased from Fine Science (Korea). Au pellets (99.99 %) were purchased from iTASCO (Korea). NAP-5 column (Sephadex G-25 DNA grade) was purchased from GE Healthcare (UK). Dithiothreitol (DTT) and HAuCl_4_ were purchased from Sigma-Aldrich (USA). T4 DNA ligase and 10× ligase buffer were purchased from Promega (USA). phi29 DNA polymerase and 10× phi29 DNA polymerase buffer were purchased from Lucigen (USA). Deoxyribonucleotide triphosphate (dNTP) mix was purchased from Epicentre (USA).

### Synthesis of Closed Circular DNA

For the fabrication of closed circular DNA, the template DNA and target DNA were mixed at the final concentration of 2.5 μM. Then, the mixture was heated at 95 °C for 2 min and gradually cooled to 25 °C for 1 h using a PCR thermal cycler (Bio-Rad, USA). After annealing, T4 DNA ligase (0.03 U μL^−1^) and ligase buffer (30 mM Tris-HCl (pH 7.8), 10 mM MgCl_2_, 10 mM DTT, and 1 mM adenosine triphosphate) were added, and the reaction solution was incubated overnight at room temperature.

### Hybridization of Primer DNA and pri-cir DNA

To reduce disulfide linkage in the primer DNA, the primer DNA was incubated for 2 h at room temperature with 0.2 M DTT and 0.18 M phosphate buffer (pH 8.0). After incubation, the primer DNA was purified using the NAP-5 column. The freshly cleaved primer DNA was mixed with an equal amount of closed circular DNA (final concentration of 1.25 μM) and incubated at room temperature for 1 h.

### Preparation of DNA-Functionalized Au Electrodes

The substrates were cleaned with an ultrasonic cleaner (Branson Ultrasonics Co., USA) in acetone, deionized water, and isopropyl alcohol for 10 min. Then, 30 nm of Au as electrodes was deposited on the substrates by thermal evaporation under 5 × 10^−7^ Torr using a shadow mask at a deposition rate of 0.1–0.2 Å/s. For the functionalization of Au electrodes with pri-cir DNA, 1 μM of pri-cir DNA was introduced on a Au electrode (gap between Au electrodes with a distance of 100 or 200 μm) with pH 8.0 phosphate buffer (final concentration of 10 mM) and 1 M NaCl and incubated overnight at room temperature. After incubation, the electrodes were washed three times with nuclease-free water.

### Analysis of the Surface of a Biosensor Covered with DNA

RCA was performed at 30 °C for 20 h, following the addition of phi29 DNA polymerase (1 U μL^−1^), phi29 reaction buffer (100 mM Tris-HCl (pH 7.5), 20 mM MgCl_2_, 20 mM (NH_4_)_2_SO_4_, and 8 mM DTT), and dNTPs (2 mM) to the electrodes. For efficient generation of DNA, multi-primer was also introduced at the beginning of RCA. After the RCA process, the electrodes were washed with nuclease-free water. For the characterization of the electrodes, an atomic force microscope (AFM) NX-10 (Park Systems Corp., Korea) was used in non-contact mode with a non-contact cantilever (PPP-NCHR, Nanosensors, Switzerland). XEI software (Park Systems Corp., Korea) was used for the analysis of the AFM images.

### Detection Procedure

After RCA, HAuCl_4_ (0.5–50 μM) was dropped on the Au electrodes (gap width 100 μm) and incubated overnight at room temperature. Then, the Au electrodes were washed three times with nuclease-free water and dried. For the measurement of electrical signal, the Agilent B2900A precision source/measurement unit (Keysight Technologies) was used, and the Agilent B2900A quick I/V measurement software was employed for data acquisition. The I-V data was obtained by varying voltages from 0 to 40 V. The resistance was calculated from the reciprocal of the slope of the current-voltage characteristic curves. For the colorimetric detection, the Au electrode with a 200-μm gap was used.

## Results and Discussion

**Fig. 1:**
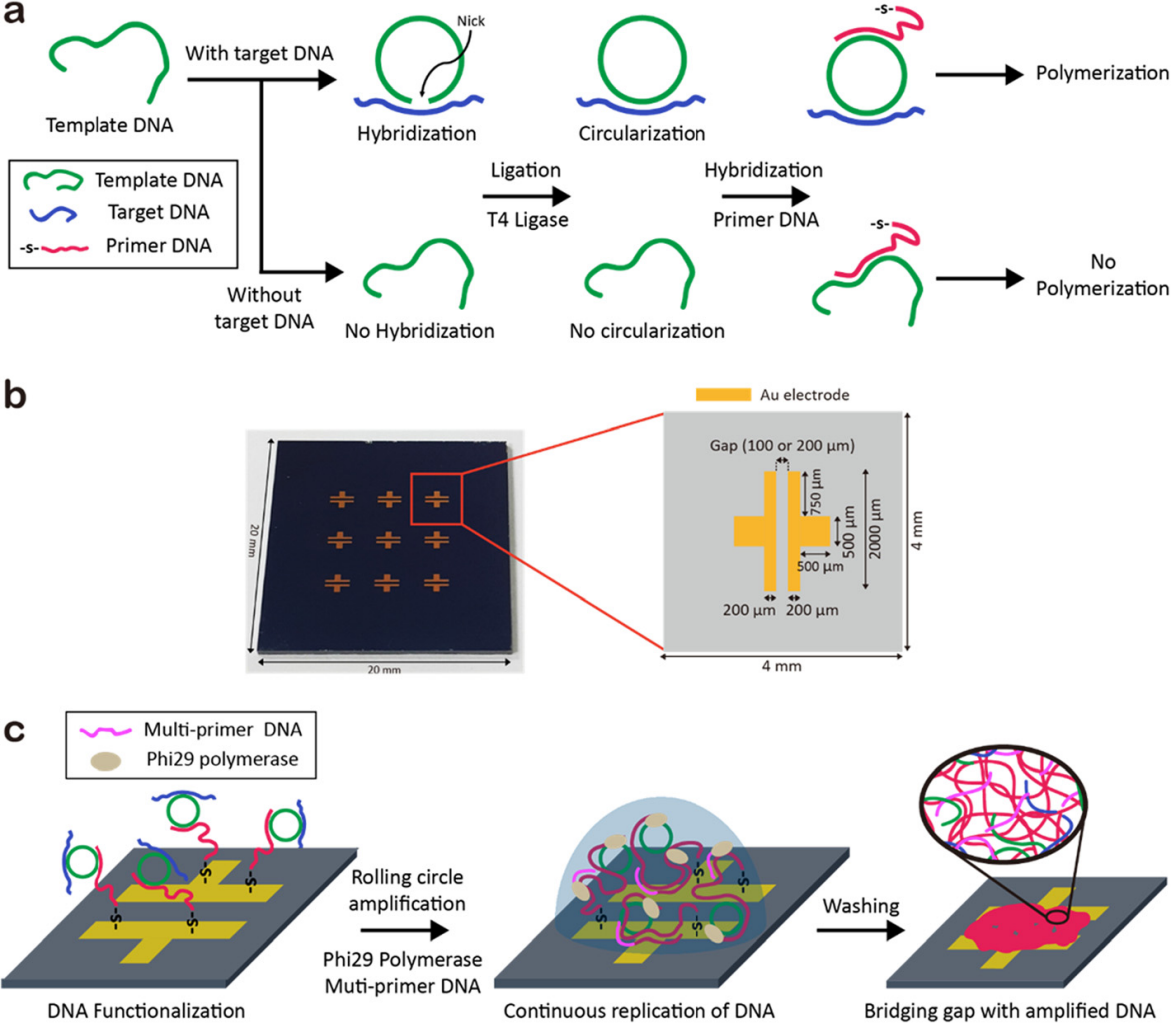
Schematic illustration of pathogen DNA detection on Au electrodes. **a** Synthesis of the closed circular DNA in the presence of target DNA (*top*). In the absence of target DNA, no circularization occurred (*bottom*). Template DNA strands in both cases are introduced with thiolated primer DNA to form hybridization. **b** Digital camera image (*left*) and detailed illustration (*right*) of the Au electrodes on Si/SiO_2_ substrate. **c** RCA process on Au electrodes with multi-primer DNA (*purple*). The amplified DNA strands fill the gap between the two electrodes

Circularization of the template DNA is one of the prerequisites to enable RCA reaction. In our system, the 5′ and 3′ ends of the template DNA were designed to hybridize with the middle region of the target DNA, leaving both ends of the target DNA as flexible overhangs. Therefore, the template DNA can be circularized when introduced with the target DNA (Fig. 1a). Then, a nick between the 5′ and 3′ ends of the template DNA is covalently linked by following a ligation process. In the absence of the target DNA, in contrast, circularization of the template DNA cannot be carried out. Thus, continual elongation of DNA is observed only in the presence of the target DNA.

Another requirement for carrying out RCA is hybridization between the closed circular DNA and primer DNA that functions as an initiation site of DNA polymerase. In our system, thiolated primer DNA was used for the immobilization of pri-cir DNA to the surface of Au electrodes. Prior to hybridization of the primer DNA with the circular DNA, disulfide linkage between the primer DNA strands were reduced with DTT. Then, the deprotected primer DNA was purified with a desalting column for the removal of an excess DTT which might hinder the functionalization of the Au electrodes with DNA strands. The resulting freshly cleaved primer DNA was introduced to the circular DNA for the production of pri-cir DNA.

For the electrical detection system, two Au electrodes with the distances of 100 or 200 μm were prepared on a Si/SiO_2_ substrate (Fig. 1b). The pri-cir DNA was then deposited and allowed to be immobilized on the surface of each Au electrode as demonstrated in Fig. 1c. After the immobilization of DNA on the Au electrodes through thiol-gold interaction, RCA is proceeded by the addition of phi29 DNA polymerase. In the presence of the target DNA, the template DNA is replicated via RCA and the products bridge the gap between two electrodes. Importantly, multi-primer DNA was also introduced to RCA reaction for the efficient amplification of DNA [29]. On the other hand, the gap between the two electrodes remained uncoated in the absence of the target DNA because RCA process could not be carried out.

**Fig. 2:**
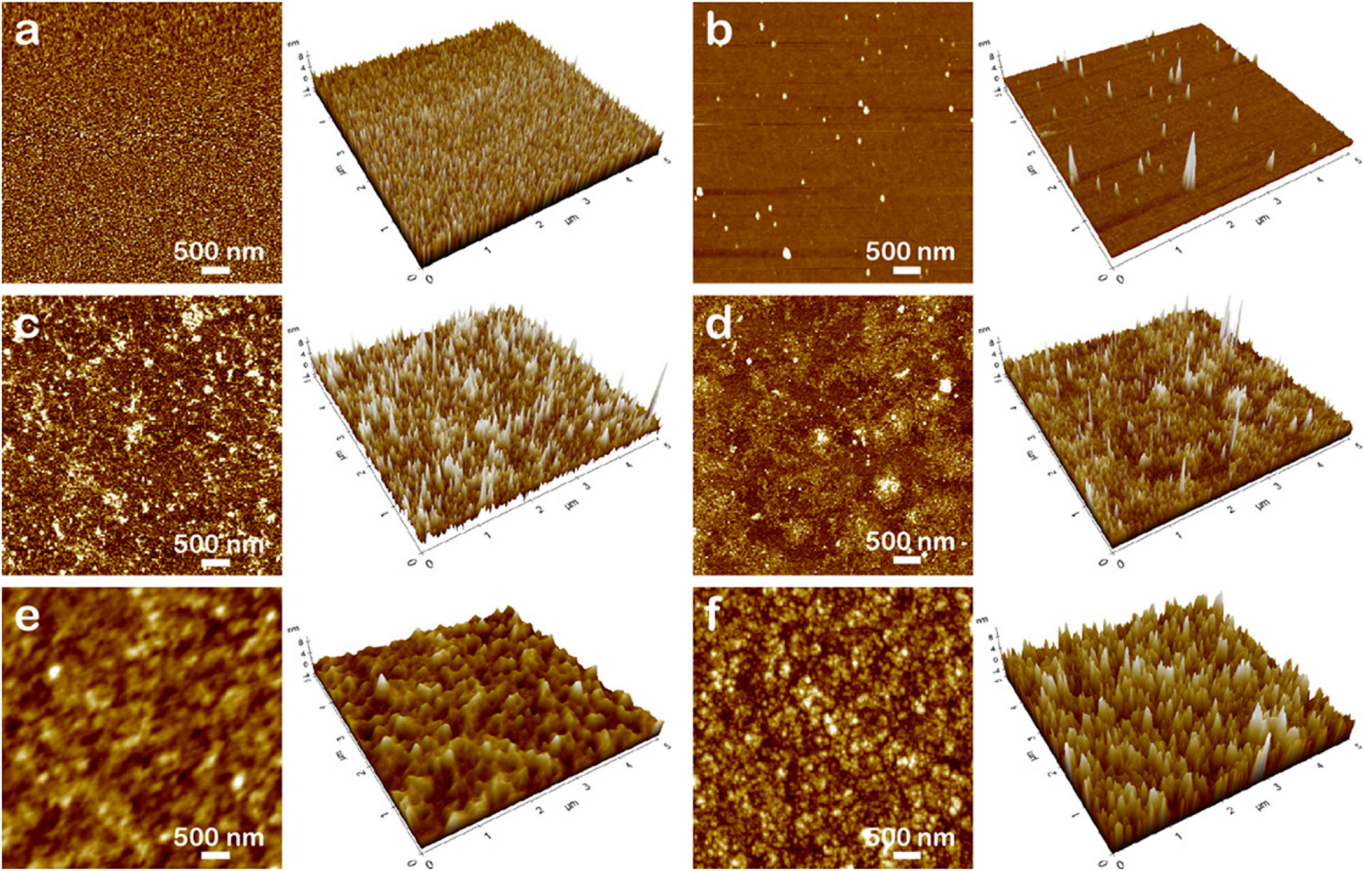
AFM images of the surfaces of Au electrodes and the gap between the electrodes. The surface of the Au electrodes (**a**, **c**, **e**) and the gap (**b**, **d**, **f**) between the electrodes (**a**, **b** the untreated; **c**, **d** treated with 0.05 μM of the target DNA; **e**, **f** treated with 1 μM of the target DNA)

To investigate the morphological changes of the substrate before and after RCA, the electrode surface and the gap between the electrodes were analyzed with AFM as shown in Fig. 2. The surface of untreated Au electrodes is covered with homogeneous gold molecules (Fig. 2a), which corresponds to a previous research [30]. In contrast, the electrodes after RCA with the target DNA have rough surfaces due to enzymatically synthesized DNA. To be specific, the AFM image of the electrode treated with 0.05 μM of the target DNA revealed an uneven surface as a result of RCA (Fig. 2c). With the higher concentration of the target DNA, the surface became coarser (Fig. 2e), suggesting that a higher amount of DNA could be replicated from the template DNA. Also, the AFM images of the gap between the electrodes indicated that the amplified DNA had covered the gap. In particular, the gap has a flat surface without performing RCA (Fig. 2b), and the roughness of the gap surface was increased after RCA with the target DNA, indicating that the gap was covered with the amplified DNA (Fig. 2d, f).

**Fig. 3:**
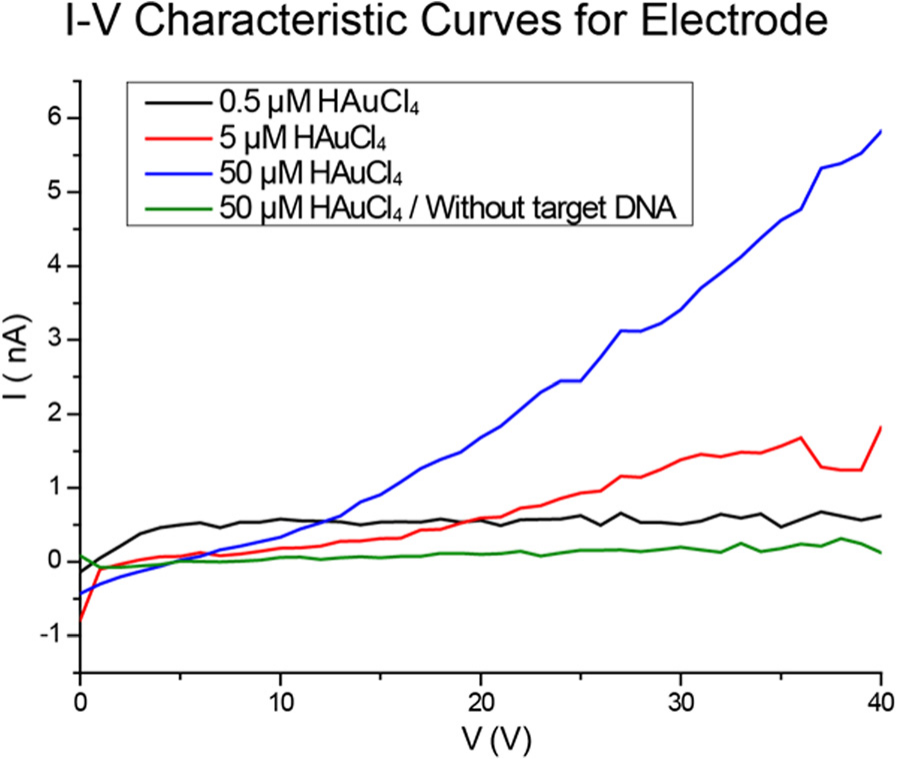
I-V characteristic curve for the detection of 0.05 μM target DNA after introduction of 0.5, 5.0, and 50 μM of HAuCl_4_. Also, the Au electrode treated with 50 μM of HAuCl_4_ after DNA amplification reaction without the target DNA was demonstrated as a control group

To detect the target DNA in an electrical manner, we further investigated electrical conductivity between the electrodes after RCA with 0.05 μM of the target DNA. To improve poor conductivity of native DNA [31], 0.5–50 μM of HAuCl_4_ was introduced to the gap-filled electrodes and allowed to be stabilized for overnight. Then, an I-V characteristic curve was obtained, changing voltages from 0 to 40 V. As shown in Fig. 3, the gap-filled electrodes treated with 50 μM of HAuCl_4_ show a gradually increasing current, while the control electrodes treated with HAuCl_4_ after DNA amplification without target DNA show no change in current. The change in electrical resistance is also demonstrated in Table 1. As the concentration of HAuCl_4_ increases, resistance between electrodes also increases due to the complexation of metal ion with DNA structures [32, 33].

**Table 1:** Changes in resistance between Au electrodes treated with various concentrations of HAuCl_4_ after RCA

	HAuCl_4_ concentration (μM)	Resistance (GΩ)
With target DNA	0.5	125
	5	20
	50	5
Without target DNA	50	143

**Fig. 4:**
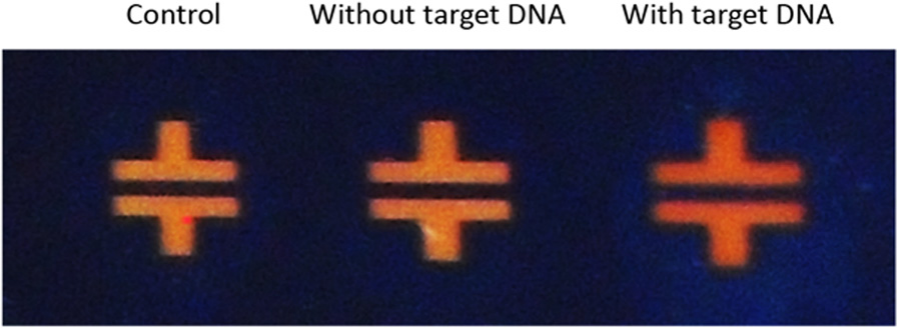
Colorimetric detection of the target DNA. Digital camera image indicates Au electrodes after RCA reaction with 1 μM of target DNA (*right*) and without target DNA (*middle*). Au electrode without any treatment was used as a control group (*left*)

In addition to the electrical signal change in the presence of the target DNA, colorimetric change was also examined after RCA (Fig. 4). As shown in Fig. 4, the color of the Au electrode changed from bright yellow to orange-red after RCA with the target DNA over 1 μM. In the absence of the target DNA, however, the color of the electrode remained unchanged after DNA amplification. In our hypothesis, this variation in optical signal is due to the interference between visible light reflected from surface of the Au electrode and DNA layer, which is in accordance with a previous research [34].

## Conclusions

In this study, the biosensor for the detection of pathogen DNA was fabricated via RCA which is one of the most powerful techniques for continuous polymerization of DNA. In the biosensor, closed circular DNAs can be generated only in the presence of the target DNA, which enables persistent polymerization of template DNA. After functionalization of the Au electrodes with the closed circular DNA, DNA strands newly synthesized by the following RCA process bridges the gap between the two electrodes. In result, the gap was connected with the amplified DNA, which was examined with AFM. Furthermore, by simply introducing HAuCl_4_ to the RCA products, electrical signal was generated without further addition of reductants for DNA metallization. In addition, the presence of 1 μM of target DNA resulted in the discoloration of the Au electrode from bright yellow to orange-red, indicating that the target DNA can be detected with the naked eye.
